# Sonar Sensor Models and Their Application to Mobile Robot Localization

**DOI:** 10.3390/s91210217

**Published:** 2009-12-17

**Authors:** Antoni Burguera, Yolanda González, Gabriel Oliver

**Affiliations:** Department de Matemàtiques i Informàtica, Universitat de les Illes Balears, Ctra. de Valldemosa Km. 7.5, E-07122 Palma de Mallorca, Spain; E-Mails: y.gonzalez@uib.es (Y.G.); goliver@uib.es (G.O.)

**Keywords:** sonar, mobile robot localization, particle filter

## Abstract

This paper presents a novel approach to mobile robot localization using sonar sensors. This approach is based on the use of particle filters. Each particle is augmented with local environment information which is updated during the mission execution. An experimental characterization of the sonar sensors used is provided in the paper. A probabilistic measurement model that takes into account the sonar uncertainties is defined according to the experimental characterization. The experimental results quantitatively evaluate the presented approach and provide a comparison with other localization strategies based on both the sonar and the laser. Some qualitative results are also provided for visual inspection.

## 1. Introduction

A crucial requirement for a mobile robot to accomplish useful long term missions is that the robot has some knowledge about its pose with respect to a fixed reference frame. The process of estimating the robot pose with respect to such fixed reference frame is known as the *localization* problem. A common approach to localize the robot is the use of *exteroceptive* sensors, such as range finders, measuring the external environment, combined with *proprioceptive* sensors, such as odometers. Exteroceptive sensor data can be correlated at subsequent robot positions to compute displacement estimates, based on initial guesses provided by proprioceptive sensors.

The quality of the pose estimates is, consequently, strongly related to the quality of the sensor measurements. That is why localization strategies usually rely on accurate sensors such as laser range scanners. Most of these sensors are able to provide thousands of readings per second with a sub degree angular resolution. Other sensors, such as standard *Time-of-Flight* (TOF) ultrasonic range finders, do not have these properties. In general terms, standard ultrasonic range finders are only able to provide tenths of readings per second and have angular resolutions one or two orders of magnitude worse than laser scanners.

However, ultrasonic range finders are still appealing in the mobile robotics community for several reasons. Their price and power consumption are better than those of laser scanners. Moreover, their basic behavior is shared with underwater sonar, which is a sensor vastly used in underwater and marine robotics. A typical underwater sonar, although being far more complex than the ultrasonic devices used in this work, can take profit of those localization techniques accounting for the ultrasonic sensor limitations.

Some researchers have demonstrated the validity of standard ultrasonic range finders, like the Polaroid ultrasonic sensors, to perform localization. For instance, Tardós *et al.* [1] used a perceptual grouping technique to identify and localize environmental features, such as corners and lines, together with robust data association to perform SLAM with sonar. Gro*β*mann *et al.* [2] confronted the sonar localization problem by means of the Hough transform and probability grids to detect walls and corners.

However, looking for features has shown to be a complex, unreliable and time consuming task due to the noisy nature of sonar data. That is why different approaches have to be adopted when using this kind of sensors. For example, Burguera *et al.* [3] defined the *sonar probabilistic Iterative Correspondence* (spIC), not requiring environmental features to be localized. They showed that scan matching localization can provide reasonably good results if sonar uncertainties are taken into account. Hernández *et al.* [4] also proposed an approach to underwater localization using sonar without requiring environmental features. One more study is by Young-Ho Choi and Se-Young Oh [5], who proposed an approach to localization using visual sonar. Although a visual sonar consists on obtaining range measurements from image data, it has comparable characteristics and poses similar problems to localization that the ultrasonic range finders.

Nowadays it is broadly accepted that probabilistic methods are the most promising ones to deal with sensor and pose uncertainties in real-time. In this context, Kalman filters are commonly used to perform localization and SLAM. However, they fail to represent ambiguities and to recover from localization failures. To confront some of these problems, Dellaert *et al.* [6] introduced the *Monte Carlo Localization* (MCL), where the probability density function is represented by a set of samples randomly drawn from it. The set of samples, which are usually called *particles*, is recursively updated by means of a method generically called *Particle Filter*.

As a nonparametric implementation of Bayes filters, particle filters have two main advantages. One is that they can approximate a wide range of probability distributions. The other is that even the most straightforward implementation of them exhibits very good results when applied to localization. Particle filters have been successfully applied to SLAM [7, 8], multi-robot localization [9] and localization given an *a priori* map using laser [10] and sonar sensors [11], among other applications. One particular study by Silver *et al.* [12] proposed the combined use of the *Iterative Closest Point* (ICP) scan matching [13] and particle filters to deal with the sparseness and low accuracy of sonar sensors in underwater environments. Although the method was only tested in simulation, this approach does not require any *a priori* map and exhibits very good results. A similar approach, tested using real sonar readings, was proposed in [14]. Although both approaches share some points in common with the research presented in this paper, they neither experimentally characterize the sonar sensor nor model it in any way.

The goal of this paper is to define, develop and experimentally evaluate an algorithm to perform MCL without *a priori* maps and using sonar sensors. The use of sonar sensors in this context is a relevant contribution of this paper, and is supported by an exhaustive experimental characterization of a widely spread sonar configuration.

More specifically, in this paper we propose the use of a probabilistic correlation technique as measurement model in a particle filter to perform mobile robot localization using sonar sensors. Each sonar reading is modeled by a bivariate Normal distribution. In order to properly model the sonar readings, an experimental characterization of the sonar sensor is performed in Section 2.. Thanks to these models, the correlation between two sets of sonar readings can be performed by means of statistical compatibility tests. The particle filter operation is described in Section 3.. Also, in this work, the particles are augmented with local environment information. This local information is updated at each time step, and allows the localization process to be performed without any *a priori* map. The aim of this local environment information is to deal with the sparseness of the sets of sonar readings. In Section 4. the model definition, the correlation process and the local map construction are presented. In order to validate and measure the quality of this approach, sonar and laser data has been simultaneously gathered in different environments. Using the laser readings, a ground truth has been constructed. Then, the sonar-based particle localization is evaluated by comparing its results to the ground truth. The quantitative evaluation method, as well as the experimental results evaluating different algorithm's parameters, are provided in Section 5. The experimental results show that the proposed approach to sonar-based localization is able to provide robust and accurate robot pose estimates. Finally, Section 6. concludes the paper.

## 2. The Polaroid Sensor

### 2.1. Overview

During the 80s, Polaroid developed a TOF ultrasonic range sensor for automatic camera focusing. Different versions of these sensors appeared. The third generation was based on the 6,500 ranging module. This module had the ability to detect and report multiple echoes. Moreover, a developer's kit was released for these sensors, allowing the user to configure the different parameters, such as frequency, gain or maximum range. Because of this, the 6,500 ranging module was extensively used in mobile robotics.

Although the original Polaroid sensors are not being used by recent robotic platforms, the 6,500 series ranging module is still commonly used. The ultrasonic sensors used in this paper are those endowed in a *Pioneer 3-DX* mobile robot. They are based on the 6,500 series ranging module and a 600 series electrostatic transducer. Throughout this paper, these sensors will be referred to as the *Polaroid sensors*.

In mobile robotics, sonar sensors are commonly used to detect objects which are in the same plane that the sensor itself. This idea will be referred to as the *2D assumption*. Thus, only the cross section of the sonar beam over the sensor plane is going to be considered in this paper. Also, it is usual to mount the sonar sensors so that their acoustic axis is parallel to the floor. In this case, under the mentioned 2D assumption, the vertical expansion rate of the sonar beam is not taken into account.

Being endowed on a specific robotic platform, some of the sensor capabilities are not accessible to the user while some other are limited because of the robot configuration. For example, the multiple echo capability is not accessible by our mobile robot. Moreover, although a firing rate of 40 ms is possible, only 2 of the 16 available sensors are accessed every 40 ms. The details of our specific robotic platform are provided in [15]. Those details of Polaroid sensors not depending on our specific robot platform are provided next.

### 2.2. Theoretical Model

**Figure 1.**
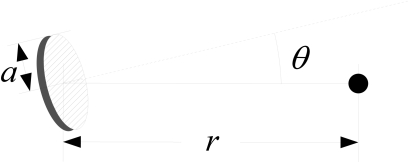

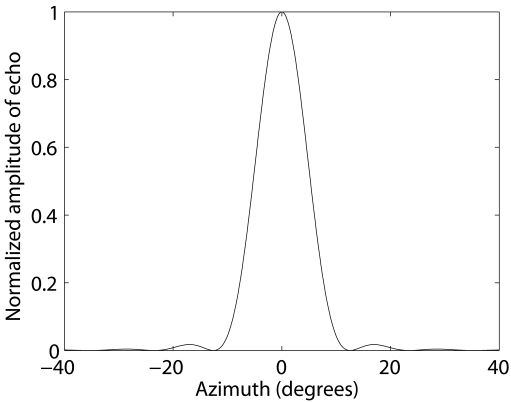

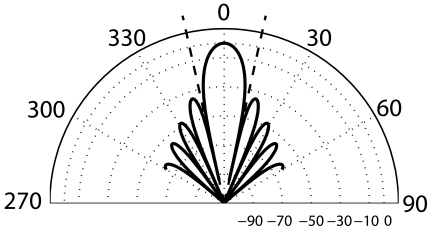

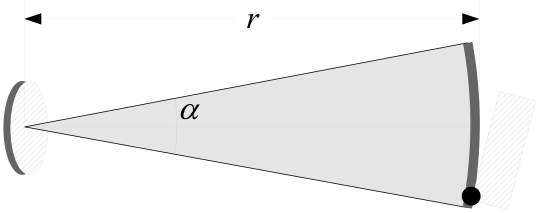
(a) Position of an object with respect to the sensor in polar coordinates. (b) Normalized sound pressure as a function of the azimuth. (c) Polar representation of the sound pressure level. The azimuth is expressed in degrees and the SPL in dB. (d) The wedge model.

Let us introduce some basic notation. If the 2D assumption is performed, the position of an object with respect to the sensor can be denoted by the polar coordinates (*r, θ*), *r* being the distance to the sensor and *θ* the orientation with respect to the sonar acoustic axis, as illustrated in Figure 1a. The angle *θ* is also named the *bearing* or the *azimuth*. A sonar sensor will be characterized by the frequency *f* and the wavelength *λ* of the emitted ultrasonic pulse, and by the radius *a* of the transducer.

The term *sound pressure*, at a given point and time, refers to the difference between the pressure of the environment outside the sound wave and the pressure found within the sound wave. The sound pressure has been used by different researchers to evaluate the sensitivity of an ultrasonic sensor. For example, Kleeman and Kuc [16] state that, under the 2D assumption, for an object located at (*r, θ*) the detected echo pressure referenced to the receiver output is given by the expression

(1) $$P(r,\theta )=\frac{\beta fa^{4}}{r^{2}}{\left(\frac{2J_{1}(ka\sin(\theta ))}{ka\sin(\theta )}\right)}^{2}$$

where *β* is a proportionality constant used to include parameters that can not be controlled by design, *k* = 2*π/λ* and *J*_1_ is the Bessel function of the first kind of order 1. Let us parametrize the previous equation for the Polaroid sensors used in this paper. According to the sensor data sheet [17], these sensors operate at a frequency *f* = 50 kHz and the transducer radius is *a* = 19.22 mm. Moreover, the temperature operating range is said to be from −30 °C to 70 °C. Let us assume a temperature of 20 °C, which is at the center of the temperature operating range. The speed of sound at this temperature is 343.21m/s. Thus, given the frequency *f* and the speed of sound, it is easy to see that *λ* = 6.86 mm.

Now, let us focus on the dependence of the echo pressure on the azimuth, assuming a constant value for *r*. Figure 1b shows the evaluation of Equation 1 with respect to the azimuth. The curve has been scaled to values between 0 and 1, to make the representation independent of the specific values of *r* and *β.* The *Sound Pressure Level* (SPL) is shown in Figure 1c in polar coordinates. The SPL expresses the relation between the sound pressure and a reference pressure as a level on a logarithmic decibel scale. The used reference pressure is the maximum pressure. Being the SPL in a logarithmic scale, some details are easier to appreciate than in the sound pressure graph.

The first thing to be noticed is that the sound pressure concentrates along the sonar acoustic axis. This area of high sensitivity is called the *main lobe*. Also, some weaker sensitivity areas appear around the azimuths ±17° and ±30°. These areas are called *side lobes*. More specifically, the main lobe is defined as the angular interval [−*θ*_0_, *θ*_0_], where *θ*_0_ is the lowest positive azimuth where Equation 1 equals to zero. In this case, as can be observed in Figure 1b, *θ*_0_ ≃ 12.5°. The interval [−*θ*_0_*, θ*_0_] lies between the dashed lines in Figure 1c.

The sonar response mainly depends on the main lobe, where the most part of the signal strength is concentrated. Because of this, it is reasonable to assume that the received echoes have been produced in the main lobe and, thus, that the azimuth of the detected object lies in [−*θ*_0_, *θ*_0_]. Consequently, there is an azimuth uncertainty of 2*θ*_0_. In the particular case of the Polaroid sensor under analysis, this theoretical azimuth uncertainty is, thus, 25°.

Taking into account the importance of the main lobe, it is common to model a sonar reading as the circular sector defined by the angular interval [−*θ*_0_, *θ*_0_] and centered at the sensor position. The sonar wedge, as defined by Moravec [18], models the sonar readings in this way. The detected object is, in consequence, assumed to lie somewhere on the boundary arc of such sector. The azimuth uncertainty, 2*θ*_0_ is then referred to as the sensor *opening*. Henceforth, the sensor opening will be denoted by *α*, thus *α* = 2*θ*_0_. This model, which is called the *wedge model*, is depicted in Figure 1d.

### 2.3. Experimental Characterization

Some studies [19] have shown that significant differences appear between the theoretical model, the sensor specifications and the real behavior of the sensor. Additionally, a significant amount of studies concerning sonar modeling in mobile robotics are based on experimental models [20–22]. Thus, we feel that an experimental evaluation of the parameters of the Polaroid sensors used in this research is necessary.

In order to build the sensor characterization, we have taken into account the following two aspects. First, although the sonar wedge model is widely used, it may be too simplistic. In consequence, the experiments are intended to find the limits of this model. Second, as the sensor is endowed on a specific robot platform, the usual way to access its measurements is by means of the robot's operating system. Accordingly, the characterization relies on reading the range information as it is provided by the robot's operating system under different conditions and, afterwards, comparing the gathered data to a ground truth. For the sake of simplicity, henceforth, the range readings provided by the robot's operating system from the Polaroid sensors will be referred to as, simply, the sensor (or the sonar) readings.

The presented analysis is based on the experimental evaluation of the following sensor parameters: resolution, minimum and maximum range, maximum angle of incidence, sensor opening and accuracy. Only the most relevant results are shown here. An exhaustive description of the evaluation procedures and the results are available in [23].

The *resolution* of a sonar is the smallest change in range that it can detect. The robot's operating system provides the range information in millimeters using an integer format. Accordingly, resolutions below one millimeter are not possible under this configuration. Taking this fact into account, a calibrated sheet was used to accurately place a wooden panel and a cardboard box at different distances from the sensor, ranging from 0m to 5 m with a 0.5 m interval. At each of these distances, the wooden panel was moved, millimeter by millimeter, a distance of 10 cm. The same process was performed using the cardboard box. Also, a similar process was done by moving, millimeter by millimeter, the sensor with respect to a stone wall. At each distance, a set of 100 sonar measurements were obtained and stored.

From these measurements, three important details have been observed. Firstly, it has been observed that the sensor is not able to measure distances up to 5 m. Secondly, we have also observed that there is a minimum detection distance. These two aspects will be discussed later. Finally, it has been observed that the smallest range that the sensor is able to detect is one millimeter. The value is constant along the whole sensor range, and is the same for all the 16 robot sensors that have been tested and for the three aforementioned materials. Accordingly the Polaroid sensor's resolution is, when it is accessed through the robot's operating system, 1 mm. This value slightly differs from the specifications, whereas the resolution is said to be 3 mm.

**Figure 2.**
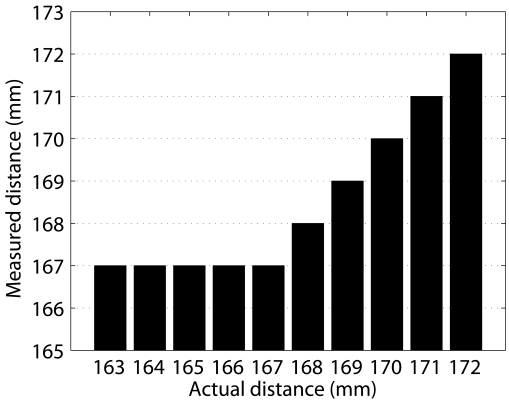

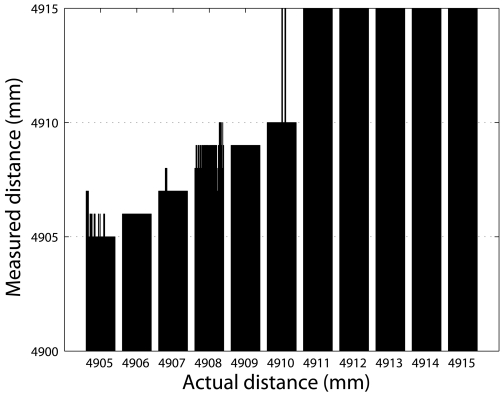
Measured distance vs. actual distance plot. For each actual distance, 100 measurements are shown. (a) Minimum range. (b) Maximum range. The resolution of 1mm can be observed in both images.

The *minimum and maximum ranges* refer to the minimum and maximum distances, respectively, at which the sensor can reliably detect an object. A first approximation of these values was obtained, as mentioned earlier, when analyzing the resolution. By placing a calibrated sheet around the previously obtained rough values, it has been observed that the minimum range is 167 mm. This value slightly differs from the one specified in the data sheet, where the minimum range is said to be 150 mm. Figure 2a exemplifies the data obtained during the minimum range determination.

The maximum range is related to the sound attenuation with distance. Our experiments show a maximum range of 4,910 mm. This value significantly differs from the sensor specification, where the maximum range is said to be 10.7 m. Taking into account this large difference it is clear that this limit is imposed by the robot's manufacturer [15]. Nevertheless, our observation also differs from the robot specification, where it is said that the maximum range is 5 m. Being this maximum range intentionally reduced by the robot's operating system, it does not depend on the material being detected and is exactly the same for all of the 16 tested sensors. Figure 2b exemplifies the data obtained during the maximum range determination.

The angle of incidence is defined as the angle between the sonar acoustic axis and the perpendicular to the detected object. The *maximum angle of incidence* refers to the maximum value for the angle of incidence producing a reliable range measurement. We have observed that this parameter is related to the material of the detected object. Broadly speaking, flat surfaces are likely to specularly reflect the most part of the ultrasonic energy and produce a low maximum angle of incidence. To the contrary, rough surfaces tend to reflect the ultrasonic pulse in scattered directions, thus providing larger values for this parameter. For example, the maximum angle of incidence with respect to an object located 1m away from the sensor is 81° if the object if a rough stone wall but only 16° if the object is a flat wooden panel.

**Figure 3.**
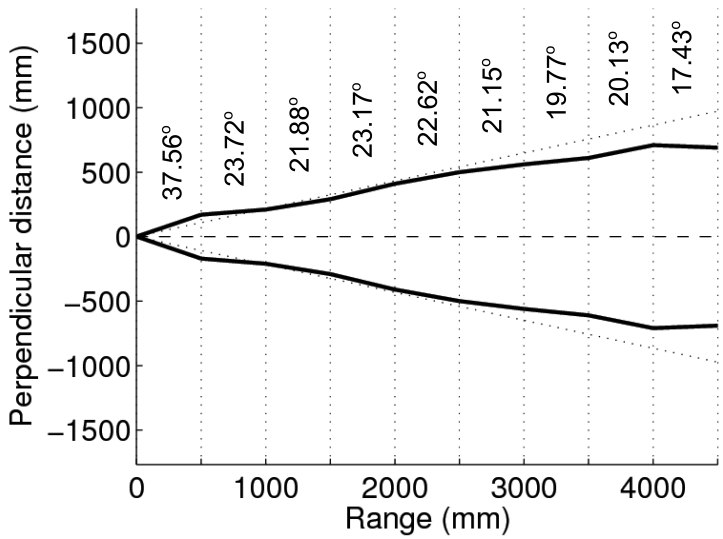

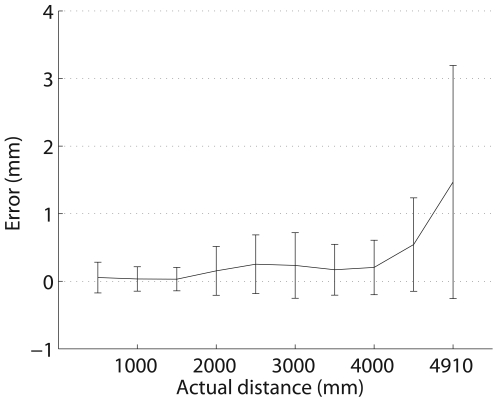
(a) Graphical representation of the sonar opening. The horizontal dashed line represents the sensor acoustic axis. The theoretical opening is shown as the two dotted lines. (b) Accuracy. Mean and standard deviation of the errors for different ranges.

As stated previously, the term *opening* refers to the azimuth interval at which the sensor is able to detect an object. The opening has been traditionally related to the main lobe of the SPL pattern. In order to take also into account the effects of the side lobes, as well as to test the limits of the sonar wedge model, the opening has been evaluated for different ranges. Being the maximum range 4,910 mm, we have measured the sensor opening for distances of 0.5, 1.5, 2, 2.5, 3, 3.5, 4 and 4.5 m. The results, as well as a graphical representation, are summarized in Figure 3a.

It can be observed how the opening is not constant. For the short range of 0.5 m, the measured opening is significantly larger than the theoretical value. This is likely to be produced by the side lobes. For large ranges the obtained opening is significantly below the theoretical value, which is probably due to the attenuation of the sound with distance.

The term *accuracy* refers to the deviation of the obtained measurements from the actual ranges. We have observed that short range readings are more accurate than the long range ones. Thus, we have measured the accuracy as a function of the range. In order to measure this parameter, sets of 500 readings each have been gathered around actual ranges of 0.5, 1, 1.5, 2, 2.5, 3, 3.5, 4, 4.5 and 4.91 m. The last range is 4.91 m, instead of 5 m, because the maximum range has been found to be 4,910 mm.

The errors between the actual and the measured ranges have been computed. The mean and the standard deviation of these errors are shown in Figure 3b. It can be observed how the mean and the standard deviation of the errors tend to increase with distance.

The obtained sensor characterization will be used in Section 4. to build the measurement model for MCL.

## 3. The Sonar Monte Carlo Localization Approach

### 3.1. Overview and Notation

Bayes filters address the problem of estimating the state *x_t_* of a dynamical system from the sensor measurements. The robot's internal knowledge about this state is usually called the *belief*, and is represented by *bel*(*x_t_*), where the subindex *t* depicts the time step. Particle filters are implementations of the Bayes filter that represent the belief by a set *X_t_* of weighted random samples, called particles, which are distributed according to the belief itself. At time *t*, the particle set is updated by means of the control vector *u_t_* and the sensor measurements *z_t_*. In the context of this paper, *u_t_* corresponds to the robot odometric data and the *z_t_* are the sonar readings. Given that this paper is centered on the localization problem, the state to be estimated is the robot pose.

The use of particle filters in localization has centered the attention of the localization community since their introduction in this context. Dellaert *et al.* [6] and Fox *et al.* [24] defined the term MCL to refer, precisely, to those localization techniques based on particle filters. The instantiation of the MCL to the sonar context will be referred to as *sonar Monte Carlo Localization* (sMCL) throughout this paper. The particularities of sMCL with respect to the standard MCL approach will also be pointed out here. Let the set of particles at time step *t* be defined as follows:

(2) $$X_{t}=\left\{\left(x_{t}^{\left[m\right]},w_{t}^{\left[m\right]},s_{t}^{\left[m\right]}\right),1\leq m\leq M\right\}$$

where *M* is the number of particles. Each $x_{t}^{\left[m\right]}$ is a concrete instantiation of the robot pose at time *t*. Under the 2D assumption *x_t_* is a three dimensional vector of the form [*x, y, θ*]*^T^*, representing the robot position and orientation. Each representing the robot position and orientation. Each $w_{t}^{\left[m\right]}$ is the particle importance factor or weight, so that, ideally, $w_{t}^{\left[m\right]}=p\left(z_{t}|x_{t}^{\left[m\right]}\right)$.

The term $s_{t}^{\left[m\right]}$ denotes the particle's local map. This local map is a short history of the most recent *k* sets of sonar readings represented with respect to a coordinate frame located at $x_{t}^{\left[m\right]}$. The use of $s_{t}^{\left[m\right]}$ and its on-line building are two of the particularities of the sMCL approach that make the localization process not dependant on *a priori* maps. A description of how this history is built and used is presented in Section 4.

Let ${\bar{x}}_{t}^{\left[m\right]}$ denote the relative robot displacement and rotation from time *t* − 1 to time *t* according to the particle *m*. Finally, let the operators ⊖ and ⊕ denote the inversion and the compounding transformations. The operator ⊕ will be used with two additions with respect to the one introduced by Smith *et al.* [25]. First, if the right-hand operand is a point, ⊕ is used to transform the reference of the point [1]. Second, if the right-hand operand is a set of points, the operator is applied individually to each point and, thus, it returns the resulting set of points.

Finally, let the superindex [1 : *M*] denote all the particles. For instance, $x_{t}^{\left[1:M\right]}$, represents the poses of all particles at time *t*, and $s_{t}^{\left[1:M\right]}$ denote all the local maps at time *t*.

**Figure 4.**
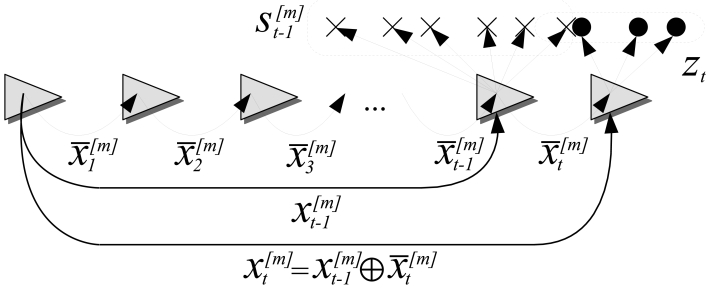
Notation used for sMCL. The triangles represent the robot at consecutive poses.

Figure 4 summarizes the notation used throughout this paper.

### 3.2. Initialization

Particle filters build the particle set *X_t_* recursively from the particle set *X_t_*_−1_ one time step earlier. Thus, it is necessary to initialize the recursion by defining *X*_0_. In general, this initialization only involves the initial population of the state space. For instance, if global localization has to be performed, the initialization usually consists on distributing the particles over the empty regions of the global map randomly according to a uniform distribution. To the contrary, if pose tracking is the goal, the whole particle set can be initialized to the starting robot pose, which is commonly assumed to be [0,0,0]*^T^*. Although our proposal lies in the pose tracking category, the initialization also involves building the initial local maps $s_{t}^{\left[m\right]}$.

In order to initialize the particle set, including the local maps, the robot has to move during *k* time steps and compute its pose using some external method such as odometry. Then, the robot pose $x_{0}^{\left[1:M\right]}$ for all particles is set to the mentioned odometric estimate. Also, during the initialization, *k* sets of sonar readings are gathered and represented with respect to $x_{0}^{\left[1:M\right]}$ based on odometric information. The history for all particles $s_{0}^{\left[1:M\right]}$ is then set to the mentioned *k* sets of sonar readings. Also, if necessary, the whole set of particles could be initialized by means of a more complex motion model, such as those described in [26].

According to the described initialization process, the value of *k*, which represents the size of each local map, also determines the duration of the initialization phase. The influence of this initial dependence on odometry on the overall particle filter operation is bounded. Different values of *k* will be experimentally tested in Section 5. Just as an example, let us consider the case *k* = 100. According to the experimental results, this is a reasonable value for the local map size. Also, our robotic platform provides a time step of 100ms. In this case, the robot has to solely rely on odometry during the first 10s of operation. In order to reduce the negative effects of this initial dependence on dead reckoning, the robot motion during the initialization process could be intentionally defined to minimize the odometric error. Nonetheless, the experimental results suggest that there is no need to force such specific motion during the initialization.

Although having to rely on dead reckoning during 10 s may seem a long time, it is important to emphasize that this initialization is needed because of the low Polaroid's sensing rate, which is, under the described robot configuration, close to 50 range measurements per second. When compared to other sensors, such as standard laser range finders, which provide thousands of readings per second, the need for a few seconds initialization time becomes clear. A similar issue is found in other studies such as [1] or [4], where more than 10 seconds are required to gather enough sonar data to perform localization.

### 3.3. The Particle Filter

When the initialization has been performed, the particle filter is ready to start. The proposed particle filter implementation is shown in Algorithm 1. Next, the particularities of this algorithm and the main differences with respect to the standard particle filtering approach are described.

Line 3 is in charge of sampling from the motion model. In general, the motion model involves sampling from the distribution *p*(*x_t_*∣*x_t_* _− 1_, *u_t_*). Different algorithms to perform such sampling exist in the literature [26]. These algorithms rely on some parameters which have to be experimentally tuned for each specific robotic platform. However, sampling from the aforementioned distribution is only necessary if global localization has to be performed. Being this study focused on pose tracking, we propose an alternative motion model which is computationally cheaper and allows the use of simpler error models. Our proposal is to sample from the posterior over local robot poses and use the compounding operator to build the final particle set. Thus, line 3 generates hypothetical robot motions ${\bar{x}}_{t}^{\left[m\right]}$ from time *t* − 1 to time *t*. It is important to emphasize that ${\bar{x}}_{t}^{\left[m\right]}$ does not depend on $x_{t-1}^{\left[m\right]}$ because it represents a relative motion, not the global robot pose. Thus, this step involves sampling from the distribution *p(x̄_t_*∣*u_t_*), where *x̄_t_* represents a robot motion from time step *t* − 1 to time step *t*. In general, the robot motion from time step *t* − 1 and time step *t* is so small that the mentioned distribution can be approximated by a Normal. In the performed experiments, the robot moved less than 2 cm between two time steps, which is a reasonably small distance to perform the Normal approximation. Moreover, in some cases, such as the one of a differential drive robot, the motion model is linear with the wheel velocities, making the Normal assumption even more feasible. Thus, our proposal for the motion model is to randomly generate the ${\bar{x}}_{t}^{\left[m\right]}$ according to a Normal *N*(*μ, P*). The mean, *μ*, can be obtained from a kinematic model of the robot. The covariance has to be obtained experimentally. However, the obtention of such covariance is easier than the obtention of the parameters involved in the algorithms to sample from *p* (*x_t_*∣*x_t_*_−1_*, u_t_*).

**Algorithm 1:** The Particle Filter algorithm.$\text{Input}:\{\begin{array}{ll} M: & \text{Number of particles}\\ X_{t-1}: & \text{Particle set}\left\{\left(x_{t-1}^{\left[1:M\right]},w_{t-1}^{\left[1:M\right]},s_{t-1}^{\left[1:M\right]}\right)\right\}\\ u_{t}: & \text{Odometry}\\ z_{t}: & \text{Sonar readings} \end{array}$$\text{Output}:\{\begin{array}{cc} X_{t}: & \text{Particle set}\left\{\left(x_{t}^{\left[1:M\right]},w_{t}^{\left[1:M\right]},s_{t}^{\left[1:M\right]}\right)\right\} \end{array}$**1****begin****2** **for***m* → 1 **to***M***do****3**  
${\bar{x}}_{t}^{\left[m\right]}\leftarrow \text{sample motion model}(u_{t});$**4**  
$w_{t}^{\left[m\right]}\leftarrow \text{measurement model}(z_{t},{\bar{x}}_{t}^{\left[m\right]},s_{t-1}^{\left[m\right]});$**5** **endfor****6** **for***m* ← 1 **to***M***do****7**  draw *i* with probability 
$w_{t}^{\left[i\right]};$**8**  
$x_{t}^{\left[i\right]}\leftarrow x_{t-1}^{\left[i\right]}⊕{\bar{x}}_{t}^{\left[i\right]};$**9**  
$$s_{t}^{\left[i\right]}\leftarrow \text{update history}(s_{t-1}^{\left[i\right]},z_{t},{\bar{x}}_{t}^{\left[i\right]});$$**10**  
$$X_{t}\leftarrow X_{t}\cup \{(x_{t}^{\left[i\right]},w_{t}^{\left[i\right]},s_{t}^{\left[i\right]})\};$$**11** **endfor****12****end**

Line 4 incorporates the measurement *z_t_* into the particle set by computing the importance factor $w_{t}^{\left[m\right]}$. The particles that best explain the current sonar readings will have best weights. In general, given the sonar readings *z_t_*, the measurement model should assign to the particle *m* the weight $p\left(z_{t}|x_{t}^{\left[m\right]}\right)$.

There are two main approaches in the literature to compute $p\left(z_{t}|x_{t}^{\left[m\right]}\right)$. On the one hand, the *Simultaneous Localization and Mapping* (SLAM) studies [7, 27], which include the map into the state vector, so that readings can be fully predicted from $x_{t}^{\left[m\right]}$. On the other hand, the approaches relying on *a priori* maps. Besides, both approaches have some problems. For example, including the maps into the state vector leads to a high dimensional state vector that makes difficult to properly populate the state space with particles [28]. This problem is magnified if the local maps are histories of readings, as the dimensionality grows significantly faster than in the approaches that make use of feature maps. Also, using an *a priori* map drastically reduces the autonomy of the robot because it can only be deployed in previously known environments.

Our proposal is to approximate these probabilities by measuring the degree of matching between the current readings and the particles' local maps. As the local maps are not included into the state vector, the problem of high dimensional state spaces does not appear. In other words, we approximate the weights by $p\left(z_{t}|{\bar{x}}_{t}^{\left[m\right]},s_{t}^{\left[m\right]}\right)$. It is important to emphasize that the global robot pose $x_{t}^{\left[m\right]}$ is not necessary and the local pose ${\bar{x}}_{t}^{\left[m\right]}$ can be used instead because the maps are represented with respect to a coordinate frame which is local to each particle. The method to approximate this probability based on the provided sonar experimental characterization is described in Section 4.

Line 7 is in charge of the resampling. At this point, the algorithm draws with replacement *M* particles. The probability of drawing each particle is its importance factor. Broadly speaking, during the resampling, the particles with good weights have higher probability to remain in the particle set. In this paper, the low variance sampling algorithm has been chosen to implement the resampling step. This is a well known algorithm whose description can be found elsewhere [26].

As stated previously, our motion model provides local robot poses. Thus, an additional step is required in order to update the global robot poses held by the particle set. This is accomplished in line 8 by compounding the global robot pose at time step *t* − 1 with the relative motion from time step *t* − 1 to time step *t*. In other words, the pose of each particle with respect to a fixed coordinate frame is updated in this line. Taking into account the initialization process, this means that this line is in charge of computing the pose of each particle with respect to the robot pose when the mission started.

Line 9 incorporates the current sonar measurements into the local maps. In order to do this, the robot motion ${\bar{x}}_{t}^{\left[m\right]}$ is used. Thus, the stored local maps are different depending on the trajectory followed by each particle. This line is also in charge of neglecting the oldest information in each local map so that its size remains constant. Moreover, all the readings are represented with respect to $x_{t}^{\left[m\right]}$ in line 9. Thanks to this last step, the measurement model does not depend on the global robot pose. A detailed description of this process is provided in Section 4. Finally, the new particle set *X_t_* is constructed in line 10.

**Figure 5.**
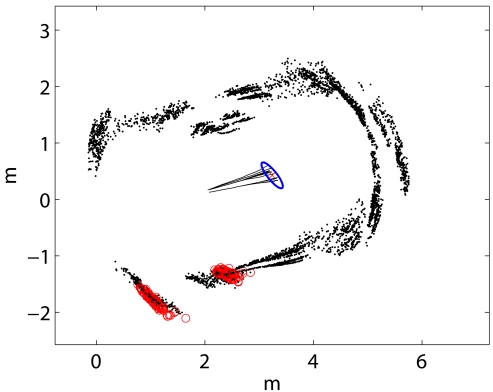

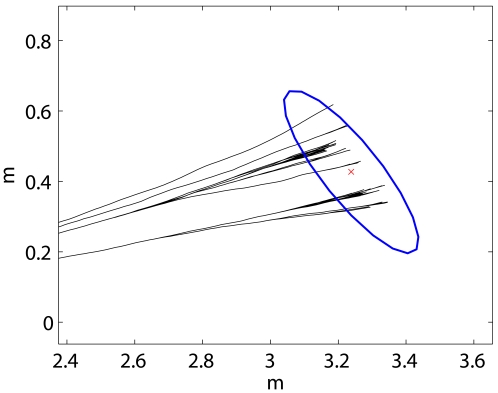
(a) Example of the particle filter operation. The local maps $s_{t}^{\left[1:M\right]}$ are depicted with black dots. The circles over the local maps represent the current readings *z_t_* according to each particle. The black lines on the center represent a short history of the robot poses in $x_{t}^{\left[m\right]}$. These trajectories are not part of the particle filter, and are only depicted for clarity purposes. Finally, the ellipse represents the 99% confidence interval corresponding to the Gaussian approximation. (b) Detail of the Gaussian approximation.

Figure 5a illustrates the different components of the presented particle filter algorithm during a real robot operation. In order to provide a clear representation, the black lines on the center of the image represent a short history of the robot poses for each particle. The specific $x_{t}^{\left[1:M\right]}$ correspond to the right end points of the mentioned lines. The black points represent the local maps. Being the robot pose values $x_{t}^{\left[1:M\right]}$ roughly divided in two groups, the local maps seem to draw a double wall. The circles represent the current readings *z_t_* according to each particle.

During the mission execution, it may be necessary not only to execute the particle filter algorithm but also to perform a *density extraction*. The density extraction consists on obtaining a continuous representation of the posterior over the robot poses instead of the discrete, sample based, representation held by the particle filter. In some cases it is sufficient to obtain the most probable robot pose instead of the full probability distribution.

Although the presented pose tracking approach may lead to multimodal distributions, we have experimentally observed that the Gaussian approximation does not introduce significant errors. Consequently, our proposal is to perform this type of density extraction when needed. Figure 5b shows an example of Gaussian approximation during one of the experiments conducted in this paper. If more accurate representations of the belief are necessary, some other density extraction techniques, such as K-means, histograms or kernel density estimation techniques should be used.

## 4. The Measurement Model

### 4.1. The Probabilistic Sonar Model

The measurement model is in charge of computing the importance factors of the particles. In particle filter localization, the importance factor represents the likelihood of having the current set of readings *z_t_* at the robot pose $x_{t}^{\left[m\right]}$. This dependence on the absolute robot pose is useful if an *a priori* global map is available, because the range readings can be matched against the global map using the absolute robot pose.

One of the advantages of the presented approach is that it does not require any *a priori* global map. Instead, each particle updates a small local map. The local map $s_{t-1}^{\left[m\right]}$ is represented with respect to $x_{t-1}^{\left[m\right]}$. Moreover, our implementation generates ${\bar{x}}_{t}^{\left[m\right]}$, which represents a relative motion between $x_{t-1}^{\left[m\right]}$ and $x_{t}^{\left[m\right]}$. Consequently, by matching ${\bar{x}}_{t}^{\left[m\right]}⊕z_{t}$ against $s_{t-1}^{\left[m\right]}$, the particle weight can be computed as an approximation of $p\left(z_{t}|{\bar{x}}_{t}^{\left[m\right]},s_{t-1}^{\left[m\right]}\right)$. Differently speaking, the idea is to evaluate the degree of matching between the current set of readings and each stored map. This idea is similar to scan matching [3, 13], where two consecutive range scans are matched to find the relative motion between them.

The proposal in this paper models the sonar readings by Normal distributions. Then, statistical compatibility tests are used to compute the degree of matching between the current sonar readings and each of the local maps.

The sonar reading provided by the *i* – *th* sonar sensor at time step *t* is modeled by the Normal distribution $r_{t}^{i}=N({\hat{r}}_{t}^{i},P_{t}^{i})$. The mean vector is ${\hat{r}}_{t}^{i}={\left[r,0\right]}^{T}$, being *r* the range measure provided by the sonar. The covariance is defined as

(3) $$P_{t}^{i}=\left[\begin{array}{cc} \sigma_{xx}^{2}(r) & 0\\ 0 & \sigma_{yy}^{2}(r) \end{array}\right]$$

where *σ_xx_*(*r*) and *σ_yy_(r)* model the range and angular uncertainties respectively. The angular uncertainty is related to the sonar opening, so that $\sigma_{yy}(r)=\frac{r}{2}\tan\left(\frac{\alpha (r)}{2}\right)$. The term *α*, which represents the opening, is written here as a function of *r* because, as shown in Section 2., the opening changes with distance. Consequently, *α(r)* has to be computed by means of the values provided in Figure 3a. For example, for a range between 1.5 m and 2 m, the opening *α(r)* should be 23.17°. Obviously, the values for *α(r)* could also be interpolated from the experimental data if necessary.

The values of *σ_xx_(r)*, representing the range uncertainty, can be obtained in a similar way from the accuracy shown in Figure 3b. The accuracy was defined as the deviation from the sonar readings to the real range, thus, the accuracy actually represents the range uncertainty.

**Figure 6.**
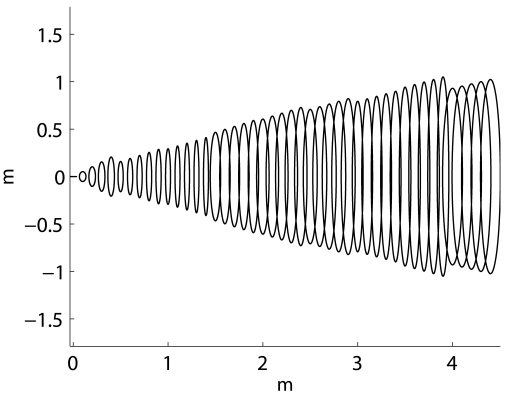

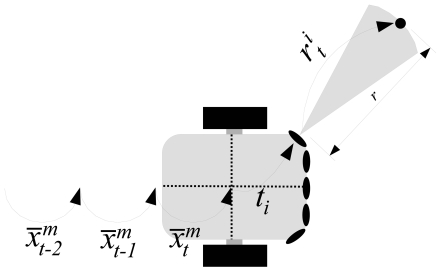
(a) The proposed sonar model. The ellipses represent the 99% confidence interval of the Normal distributions modeling the readings for different ranges. (b) Relation between the sensor models. The circular sector represents the sonar beam. The dashed cross is the robot reference frame.

Figure 6a exemplifies the proposed model. The Normal distributions corresponding to different ranges have been computed as described, and the 99% confidence ellipses drawn. The range uncertainty has been increased by a factor of 50 to provide a clear representation. The meaning of the model becomes clear when it is compared to Figure 3a.

For a given particle *m*, the robot motion from time step *t* − 1*to* time step *t* is modeled by the Normal distribution ${\bar{x}}_{t}^{m}=N({\bar{x}}_{t}^{m},P)$. The mean of this distribution is, precisely, the motion generated by the motion model in line 3 of Algorithm 1. The covariance has to be defined so that ${\bar{x}}_{t}^{m}$ is a good approximation of the distribution *p(x̄_t_*∣*u_t_)* used in the motion model. If the motion model also makes use of Normal distributions, as it was described previously, the same covariance can be used here.

Finally, the relative position of the sonar sensor *i* with respect to the robot reference frame is represented by *t_i_*. This relative position do not change over time, and is perfectly known. Figure 6b depicts the relation between the described models.

### 4.2. Building the Local Maps

As stated previously, the particles are weighted by matching the current set of readings *z_t_* against the stored readings $s_{t-1}^{\left[m\right]}$. The former set has been gathered at the current robot pose whereas the latter one is represented with respect to the robot pose one time step earlier. In order to match them, both sets have to be represented with respect to the same coordinate frame. The current set *z_t_* has less readings than the history. Accordingly, from a computational point of view it is preferable to transform the points in *z_t_*. The set of readings *z_t_* is built as follows

(4) $$z_{t}=\{t_{i}⊕r_{t}^{i},\forall i\in VS_{t}\}$$

where *VS_t_* is the set of sonar sensors that have generated a reading during time step *t*. Each item in *z_t_* will be denoted $z_{t}^{i}=N({\hat{z}}_{t}^{i},Q_{t}^{i})$, meaning that it was gathered at time *t* by the *i* – *th* sonar sensor.

Let *S_new_* be defined as the set of readings in *z_t_* represented with respect to the coordinate frame of $s_{t-1}^{\left[m\right]}$ (see Figure 4) using the motion ${\bar{x}}_{t}^{m}$ proposed by the particle:

(5) $$S_{\text{new}}={\bar{x}}_{t}^{m}⊕z_{t}=\{{\bar{x}}_{t}^{m}⊕z_{j}^{i},\forall i\in VS_{t}\}$$

Each item in *S_new_* will be denoted by $p_{t}^{i}$, meaning that it has been generated from $z_{t}^{i}$.

To ease notation, let us denote by *S_old_* the history $s_{t-1}^{\left[m\right]}$. It was stated previously that all the readings in *S_old_* are represented with respect to the robot pose at time step *t* − 1. Using the defined sensor models, this is accomplished as follows:

(6) $$\begin{array}{lll} S_{\text{old}} & = & \{z_{t-1},\\ & & ⊖{\bar{x}}_{t-1}^{m}⊕z_{t-2},\\ & & ⊖{\bar{x}}_{t-1}^{m}⊕(⊖{\bar{x}}_{t-2}^{m})z_{t-3},\ldots ,\\ & & ⊖{\bar{x}}_{t-1}^{m}⊕\ldots ⊕(⊖{\bar{x}}_{t-k+1}^{m})z_{t-k}\} \end{array}$$

where *k* is the history size. By observing the previous equation it is easy to see that it is not necessary to perform these computations at each time step. Instead, line 9 of Algorithm 1 computes $s_{t}^{\left[m\right]}$ from $s_{t-1}^{\left[m\right]}$ as follows:

(7) $$s_{t}^{\left[m\right]}=((⊖{\bar{x}}_{t}^{\left[m\right]})⊕s_{t-1}^{\left[m\right]})\cup z_{t}$$

First, the readings in $s_{t-1}^{\left[m\right]}$ are represented with respect to the coordinate frame of $x_{t}^{\left[m\right]}$ by compounding them with $⊖{\bar{x}}_{t}^{\left[m\right]}$. Then, the new set of readings *z_t_* is added. Finally, although not being represented in Equation 7, the oldest readings in the resulting set have to be deleted so that the size of the local maps remains constant along the whole mission execution. Thanks to the recursive update, the computational effort required to build *S_old_* is significantly reduced. Moreover, it is not necessary to store a history of the robot motions. A special situation arises at the beginning of the particle filter operation, when *t* < *k*. In that case, part of the readings in *S_old_* come from the initialization process.

**Figure 7.**
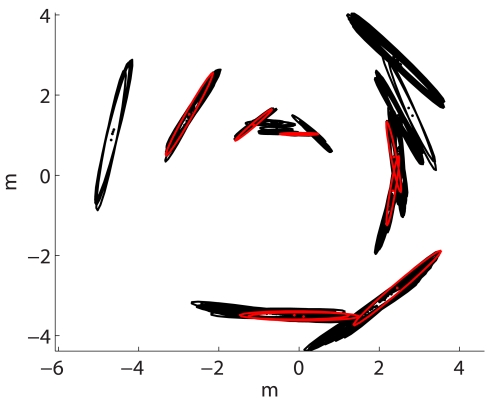
Example of sonar readings *S_new_*, in red, and *S_old_*, in black. The ellipses depict the 99% confidence interval of the associated Normal distributions.

Figure 7 shows an example of *S_new_* and *S_old_* built as described. It can be observed how the obtained Normal distributions are well suited to model the sonar uncertainties, especially the angular one.

### 4.3. The Probabilistic Approach

There exist many algorithms to match sets of range readings [13, 29, 30]. Most of them follow the structure proposed by the ICP algorithm [13]. One important step in this algorithm is the establishment of *correspondences* between readings in two consecutive sensor scans. A correspondence is an association between a reading in *S_new_* and a reading in *S_old_*. Correspondences are commonly established in two steps. First, *S_new_* is represented with respect to the coordinate frame of *S_old_* by using the current pose estimate. Then, correspondences are defined according to a proximity criteria: a reading in *S_new_* is associated with the closest one in *S_old_*. The proximity criteria usually relies on the Euclidean distance. When it is built, a set of correspondences gives information about the degree of matching between the two sets of readings.

The proposed measurement model is based on matching the current set of readings against the local maps. However, instead of using the Euclidean distance, this method proposes the use of the Normal distributions in *S_new_* and *S_old_* to establish correspondences only for those readings which are statistically compatible. Then, the degree of matching can be computed from the overall statistical compatibility.

Let *q_j_* = *N(q̂_j_, P_j_)* be a reading in *S_old_*. To decide whether $p_{t}^{i}\in S_{\text{new}}$ and *q_j_* are statistically compatible, the squared Mahalanobis distance is used:

(8) $$D^{2}(p_{t}^{i},q_{j})={\left(p_{t}^{i}-q_{j}\right)}^{T}P^{-1}(p_{t}^{i}-q_{j})$$

where *P* is the covariance matrix associated to $p_{t}^{i}-q_{j}$. Equation 5 shows that each $p_{t}^{i}$ in *S_new_* is the result of compounding ${\bar{x}}_{t}^{m}$ with $z_{t}^{i}$. Thus, to compute the covariance, the compounding operation is linearized around ${\bar{x}}_{t}^{\left[m\right]}$ and ${\hat{z}}_{t}^{i}$. As defined in [1], let *J*_1⊕_ and *J*_2⊕_ denote the Jacobian matrices of the compounding operator with respect to the first and second operands respectively, evaluated at the mentioned linearization points. Then, the covariance can be computed as follows:

(9) $$P=J_{1⊕}P_{t}^{\left[m\right]}J_{1⊕}^{T}+J_{2⊕}Q_{t}^{i}J_{2⊕}^{T}+P_{j}$$

The Mahalanobis distance in Equation 8 is, under Gaussian assumption, a chi-squared distribution with 2 degrees of freedom. Thus, $p_{t}^{i}$ and *q_j_* are compatible if and only if $D^{2}(p_{t}^{i},q_{j})<\chi_{2,\beta }^{2}$, where *β* is the desired confidence level. Accordingly, for each $p_{t}^{i}$, the set of compatible points in *S_old_* is built. Among them, the corresponding point *q_j_* is selected as the one which is closer to $p_{t}^{i}$ in the Mahalanobis sense. That is, the set of correspondences is defined as follows:

(10) $$C=\{(p_{t}^{i},q_{j})\forall p_{t}^{i}\in S_{\text{new}}|q_{j}\in S_{\text{old}},q_{j}=\arg\min D^{2}(p_{t}^{i},q_{j}),D^{2}(p_{t}^{i},q_{j})\leq \chi_{2,\beta }^{2}\}$$

To ease notation, let *C* = {< *a_i_, b_i_* >, 1 ≤ *i* ≤ *n*} be the set of established correspondences between *a_i_* ∈ *S_new_* and *b_i_* ∈ *S_old_*. The sum of squared Mahalanobis distances between pairs of corresponding points, $\sum_{i=1}^{n}D^{2}(a_{i},b_{i})$, is a good indicator of the degree of matching between *S_new_* and *S_old_*: the worse the matching, the bigger the sum of distances. However, the importance factor represents the opposite idea: particles that produce better matching should have higher weights. In consequence, the importance factor for particle *m* is computed as follows:

(11) $$w_{t}^{\left[m\right]}={\left(\sum_{i=1}^{n}D^{2}(a_{i},b_{i})\right)}^{-1}$$

At this point, our approach to Monte Carlo Localization using sonar sensors has been fully defined.

## 5. Experimental Results

### 5.1. Experimental Setup

The experimental results presented in this section are aimed at evaluating the quality of the presented particle filter localization approach and to compare the two proposed measurement models, both in terms of quality and time consumption.

To evaluate the sMCL, a *Pioneer 3-DX* robot, endowed with 16 Polaroid ultrasonic range finders and a *Hokuyo URG-04LX* laser scanner has been used. The robot has moved in three different environments in our university, gathering various data sets.

Each data set contains the odometry information, the sonar range readings and the laser range readings. The laser readings have only been used to obtain ground truth pose estimates. In order to obtain such ground truth, the ICP scan matching algorithm [13] has been applied to the laser readings. The resulting ICP trajectory has been improved manually to overcome some of the ICP limitations. Finally, the wheel encoder readings have been corrupted with a small amount of Gaussian noise (*μ* = 0*m/s* and *σ*^2^ = 0.0025) to simulate worse floor conditions.

**Figure 8.**
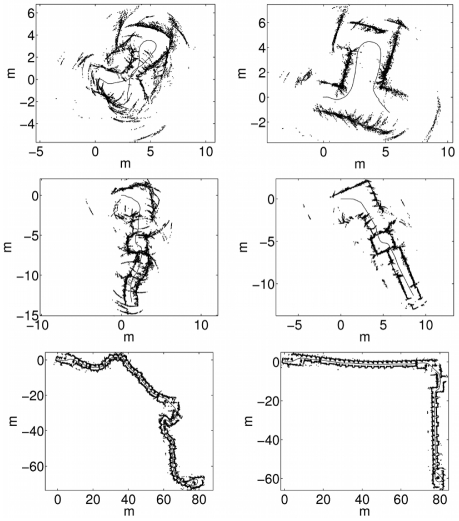
Some of the data sets using in the experiments. The left column shows the sonar readings according to the initial odometric estimates. The right column shows the sonar readings according to the ground truth trajectory.

Figure 8 shows three of the gathered data sets, each of them corresponding to a different test environment. The first one (top row) corresponds to an area with rough walls, a glass door and two wooden doors. The data sets gathered in this environment are especially problematic to perform localization due to the curved trajectory. In the second environment (middle row), the most part of the walls are covered with wooden bookshelves. The junctions between the bookshelves produce large artifacts when observed with sonar sensors, as it can be observed on the top of the images. Additionally, the small squared room between the L-turn and the corridor is problematic because localization algorithms tend to align its walls with those of the corridor, although not being actually aligned. Finally, the third environment (bottom row) corresponds to two corridors with multiple entrances to offices. The walls are rough and some wooden panels cover them in different parts. Also, there are some wooden and stone columns. The average robot speed was 5.34 cm/s in the first environment, 5.05 cm/s in the second one and 14.5 cm/s in the third one.

**Figure 9.**
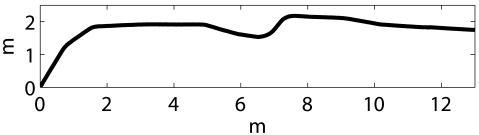

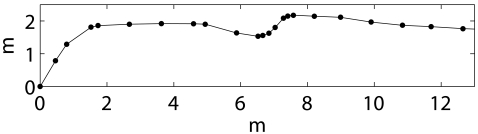
(Top) Fragment of a real trajectory. (Bottom) Polyline approximation. The dots represent the vertexes.

In order to quantitatively compare odometry and the different particle filter configurations, the following procedure has been used. First, the trajectories obtained by odometry, particle filter and the ground truth are approximated by polylines. The vertex density of each polyline increases in those regions with significant amount of robot rotation. Also, the maximum robot motion between two vertexes has been set to 1 m. This kind of approximation is useful to overcome the local perturbations in the individual motion estimates, both for odometry, particle filter and ground truth. Figure 9 exemplifies the polyline approximation.

Then, the individual edges of the trajectory being evaluated are locally compared to those of the ground truth. The Euclidean distance between their end points is used as a measure of the edge error. Finally, the edge errors for the trajectory being evaluated are summed. This sum is normalized, using the path lengths between vertexes and the number of edges, and constitutes the *trajectory error.* Due to the mentioned normalization, the errors of different trajectories can be compared. It is important to emphasize that, as a result of the mentioned procedure, the evaluation takes into account the whole trajectory, not only its end points.

The sonar-based particle localization algorithm described in Section 3. has also been implemented using a different measurement model. This different measurement model is the well known ICP error, which uses Euclidean distance to establish correspondences. Then, the probability distribution $p\left(z_{t}|{\bar{x}}_{t}^{\left[m\right]},s_{t-1}^{\left[m\right]}\right)$ is defined as the inverse of the sum of Euclidean distances between pairs of corresponding points. Thus, a well-known and widely used algorithm has also been quantitatively evaluated in the described particle filter context and compared to our approach. To distinguish between the two MCL approaches, the one described in this paper will be referred to as the *sonar probabilistic Monte Carlo Localization* (spMCL) and the one that makes use of the ICP error will be named *ICP Monte Carlo Localization* (icpMCL).

### 5.2. Evaluating the Influence of the Number of Particles

The first experiment evaluates the quality and the execution time of the algorithms with respect to the number of particles, *M*. The values of *M* that have been tested are 10 and 50, to see how the algorithm behaves with a small number of particles, and then 100, 200 and 400 particles. The particles history size has been set to *k* = 100. The trajectory error has been computed for odometry, for the icpMCL and for the spMCL.

**Figure 10.**
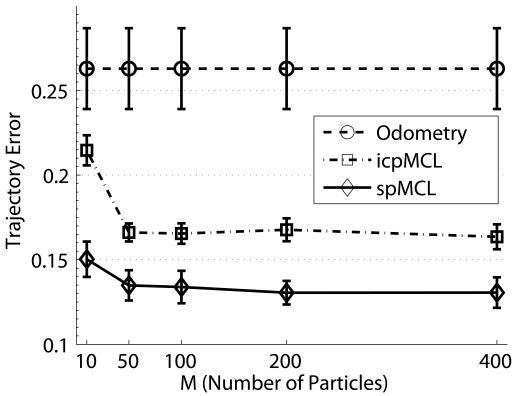

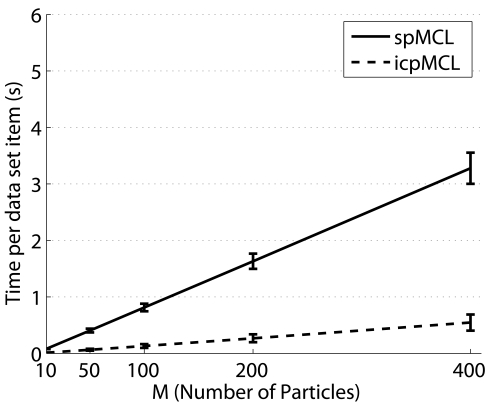
(a) Means and standard deviations of the trajectory errors. The experimental results have been obtained using different numbers of particles and setting the history size *k* to 100. (b) Means and standard deviations of the execution time per data set item on a Matlab implementation. The experimental results have been obtained using different numbers of particles and setting the history size *k* to 100.

Figure 10a depicts the mean and the standard deviation of the obtained trajectory errors for all the data sets. The graphical representation of the standard deviation has been reduced to a 20% of its real value, both for particle filters and odometry, to provide a clear representation. Also, although the odometric error does not depend on the number of particles, it has been included on the Figure as an horizontal line with constant standard deviation for comparison purposes.

The first thing to be noticed is that the two measurement models presented in this paper significantly reduce the odometric error. Also, the results provided by the spMCL are significantly better than those obtained when the ICP error is used. Even if only 10 particles are used, the spMCL provides trajectories which are, in mean, a 74.9% better than the odometric estimates and a 42.8% better than the icpMCL. If 400 particles are used, spMCL provides trajectories which are, in mean, a 101.3% better than odometry. Also, the standard deviations of the particle filter trajectories are significantly lower than those of odometry. This suggests that the quality of the particle filter estimates is barely influenced by the errors in odometry. Moreover, the reduced standard deviation also suggests that the trajectory error after the particle filter operation is similar for all of the tested environments.

The second thing to be noticed is that only a very small error reduction appears if more than 200 particles are used. This suggests that the proposed weighting methods are able to accurately select the right particles. It also suggests that using a number of particles between 100 and 200 would be a good choice, more if the execution times are taken into account.

The mean and the standard deviation of the execution times per data set item for the particle filter algorithm using each of the measurement models presented in this paper are shown in Figure 10b. It is important to emphasize that these execution times correspond to a non optimized Matlab implementation. Thus, the absolute values are meaningless, as a C++ implementation will greatly increase the execution speed. The main interest of these results is to see how the execution times evolve with the number of particles and to compare the two proposed measurement models in terms of computational requirements.

It can be observed how the execution times are strongly linear with the number of particles. The small standard deviations suggest that there is a very small dependence on the number of effective readings in each *S_old_* and *S_new_* and also a very small dependence on the odometric error and the particularities of each environment. Also, the linear relation between time and number of particles reinforces the idea that using between 100 and 200 particles is the better choice: the small improvement of using more particles does not compensate the increase in computation time.

Finally, it is clear that the icpMCL is significantly faster than spMCL in terms of the number of particles. Still, the quality of the pose estimates has to be taken into account when analyzing the time consumption. For example, using only 10 particles in spMCL provides, in mean, trajectories a 10.11% better than those provided by icpMCL when using 100 particles. Moreover, in this case, the probabilistic approach is a 59% faster than the ICP error based approach. As a matter of fact, using 10 particles in spMCL leads to better results than the icpMCL with any of the tested number of particles. Thus, when analyzing the time consumption required to achieve a certain trajectory error, the spMCL also provides significantly better results than the icpMCL.

### 5.3. Evaluating the Influence of the Local Map Size

**Figure 11.**
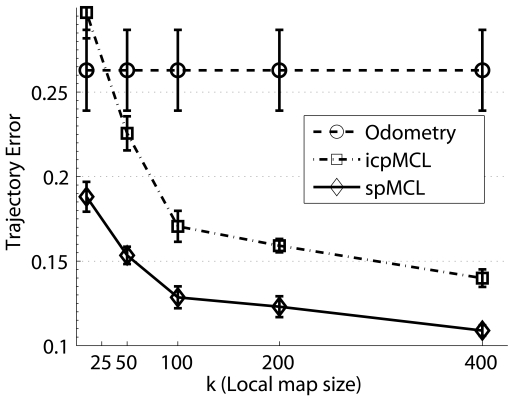

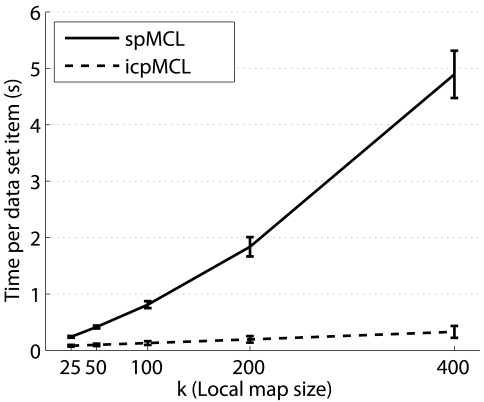
(a) Means and standard deviations of the trajectory errors. The experimental results have been obtained using different local map sizes and setting the number of particles *M* to 100. (b) Means and standard deviations of the execution time per data set item on a Matlab implementation. The experimental results have been obtained using different local map sizes and setting the number of particles *M* to 100.

The previous experiment has been performed using a local map size *k* = 100. In order to quantify the effects of using different history sizes, a second experiment has been performed. Now, the number of particles is set to 100, as it has shown to be a good choice, and the local map sizes *k* = 25, *k* = 50, *k* = 100, *k* = 200 and *k* = 400 are tested. Both the trajectory error and the execution times are measured. Figure 11a shows the mean and the standard deviation of the trajectory errors, both for odometry and the two particle filter configurations. The standard deviation has been graphically reduced to a 20% to provide a clear representation.

It can be observed that the effects of the local map size are more noticeable than those of the number of particles. For example, in the spMCL case, the trajectory error using *M* = 100 and *k* = 400 is a 19.9% lower than the trajectory error using *M* = 400 and *k* = 100. It can also be observed how the error reduction ratio significantly decreases from *k* = 100 onwards.

The icpMCL does not perform very well when low local map sizes are used. For instance, in the case *k* = 25, icpMCL produces results worse than odometry. To the contrary, even in that case, spMCL greatly improves the odometric results and provides trajectories a 57.9% better than icpMCL.

Figure 11b shows the mean and the standard deviation of the execution times per data set item, with respect to the local map size. Similarly to the previous experiment, these times correspond to a non optimized Matlab implementation.

The first thing to be noticed is related to the nonlinear relation between *k* and the execution time. Changes in the computation time should be linear with the local map size. As stated previously, the computations in the measurement model are achieved by comparing the readings in *z_t_* to the local maps. As the size of *z_t_* does not depend on the local map size, increasing the value of *k* should result in a linear increment of the execution time. However, the times shown in Figure 11b for spMCL seem to contradict this affirmation. Nonetheless, there is no contradiction. It has also to be taken into account the increasing memory requirements to store the local maps and the associated covariance matrices. The time spent by the operating system and Matlab itself for memory management is, in this case, responsible of the non linearities in the execution times.

It can also be observed that the ICP error based approach is significantly faster than spMCL with respect to the local map size. The situation in this experiment is different to the previous experiment. For instance, the computation time for spMCL using *k* = 25 is below the computation time for icpMCL using *k* = 400. However, the quality of the ICP error based for *k* = 400 is better than the one of spMCL using *k* = 10. Thus, in a hypothetical situation in which only *k* could be changed and *M* had to remain unchanged, the ICP error based approach is preferable.

Nonetheless, when taking into account both the number of particles and the local map size, spMCL provides better results. For example, the use of only 10 particles and a local map size of 100 in spMCL provides a trajectory error which is a 5.8% lower than the ICP error based approach using 100 particles and a map size of 200. Moreover, in this case, the spMCL approach is significantly faster than the icpMCL.

Overall, it is clear that spMCL is not as well suited as the icpMCL to deal with large local maps in terms of computational requirements. Still, spMCL is able to provide significantly better results with very reduced numbers of particles and small local map sizes than the icpMCL using larger particle sets.

### 5.4. Qualitative Evaluation

In order to illustrate the previous results, some data sets have been plotted according to the obtained trajectories for visual inspection. Figure 8 shows some examples of the data sets used in the experiments. The left column shows the sonar readings drawn according to the initial odometric estimates. As stated previously, these odometric estimates, which constitute the control vectors *u_t_* which have been used to fed the particle filters, are corrupted with Gaussian random noise. The right column shows the sonar readings drawn according to the ground truth trajectory.

**Figure 12.**
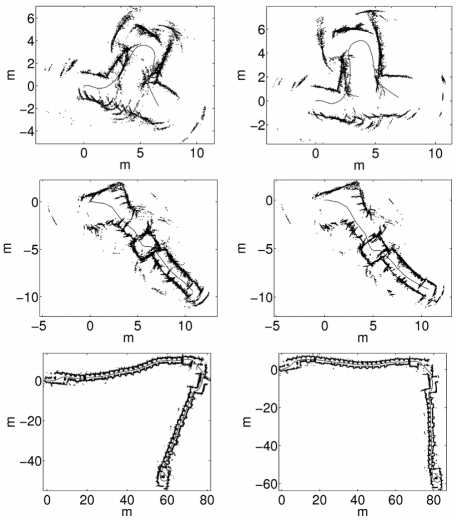
Sonar readings plotted according to the particle filter trajectories. The left column corresponds to icpMCL using 100 particles. The right column corresponds to spMCL using only 10 particles. The local map sizes are *k* = 100 in all cases.

According to the previous experiments, the spMCL with only 10 particles lead the particle filter to better results than the use of the ICP error based with much higher numbers of particles. Figure 12 illustrates this idea. The left column shows some of the results obtained when using the ICP error based measurement model with *M* = 100. The right column depicts some results obtained when using spMCL with only 10 particles.

There are some details that deserve some attention in this Figure. First, the two measurement models are able to significantly improve the odometric estimates and to provide results which are close to the ground truth. Second, the spMCL is able to match the readings more accurately than the icpMCL. This is especially clear in the second row, where the readings drawn according to the spMCL define thinner walls than those obtained by means of the ICP error based one. Finally, the results provided by spMCL provide, in general, better trajectory estimates for *M* = 10 than those provided by icpMCL using *M* = 100.

**Figure 13.**
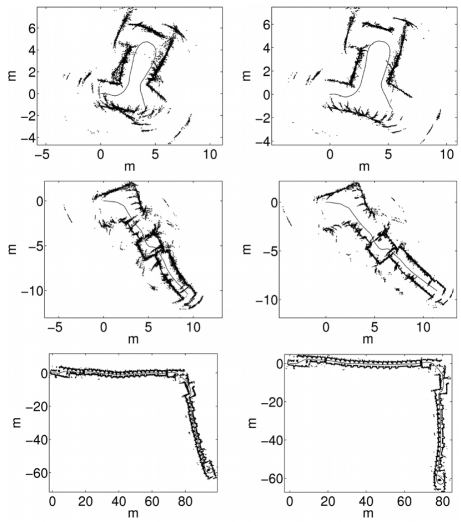
Sonar readings plotted according to the particle filter trajectories. The left column corresponds to icpMCL. The right column corresponds to spMCL. The local map sizes are *k* = 100 and the number of particles is *M* = 400 in all cases.

In Figure 13 the sonar readings have been drawn according to the two proposed measurement models using 400 particles in both cases. The left column shows the results for the ICP error based approach. The results corresponding to spMCL are presented in the right column. It can be observed that spMCL provides better results than icpMCL. Moreover, spMCL is able to generate trajectories which are very close to the ground truth.

Overall, the two sMCL approaches presented in this paper provide significant improvements in the pose estimates with respect to raw odometry. In particular, the spMCL has shown to provide trajectories which are very close to the ground truth. As stated previously, the ground truth has been obtained by manually improving the ICP pose estimates using laser readings. Moreover, the odometry estimates used in the particle filters are worse than those used for the ICP to build the ground truth. Thus, the results obtained with spMCL are comparable to those obtained with the well known ICP algorithm and laser sensors.

## 6. Conclusions

This paper is concerned to the use of sonar sensors to perform mobile robot localization. To this end, the Polaroid ultrasonic range finder has been experimentally characterized. Thanks to this characterization, parameters such as the resolution, the minimum and maximum ranges, the maximum angle of incidence, the opening or the accuracy have been obtained. Among them, the opening and the accuracy have shown to depend on the range to the detected obstacle.

Afterwards, a novel approach to mobile robot localization using sonar sensors has been presented. This approach relies on the use of particle filters. Each particle is augmented with a set of sonar readings, which is updated during the mission execution. These sets of sonar readings, which constitute the particles' local views of the environment, have two main goals. First, to overcome the sparseness of the sets of readings provided by ultrasonic range finders. Second, to avoid the need for *a priori* maps.

In order to weight the particles, a probabilistic correlation method is proposed. This method models the sonar readings as bivariate Normal distributions, allowing the use of statistical compatibility tests to evaluate the degree of matching between two sets of sonar readings. The parameters of the Normal distributions modeling the sonar readings come from the opening and the accuracy that were previously obtained.

The method has been evaluated by measuring the quality of its estimates for different numbers of particles and history sizes. Our measurement model has been compared to the well known ICP error approach, showing significantly better results. Also, the results show how the proposed sonar-based particle localization approach is well suited to deal with the sparseness and low angular resolution of sonar readings and provide good estimates of the robot pose using sonar sensors without any *a priori* map.
